# Visceral leishmaniasis: Spatiotemporal heterogeneity and drivers underlying the hotspots in Muzaffarpur, Bihar, India

**DOI:** 10.1371/journal.pntd.0006888

**Published:** 2018-12-06

**Authors:** Caroline A. Bulstra, Epke A. Le Rutte, Paritosh Malaviya, Epco C. Hasker, Luc E. Coffeng, Albert Picado, Om Prakash Singh, Marleen C. Boelaert, Sake J. de Vlas, Shyam Sundar

**Affiliations:** 1 Department of Public Health, Erasmus MC, University Medical Center Rotterdam, Rotterdam, The Netherlands; 2 Department of Public Health, Institute of Tropical Medicine, Antwerp, Belgium; 3 Department of Medicine, Institute of Medical Sciences, Banaras Hindu University, Varanasi, India; 4 ISGlobal, Barcelona Institute for Global Health, Hospital Clínic, Universitat de Barcelona, Barcelona, Spain; 5 Foundation for Innovative New Diagnostics (FIND), Geneva, Switzerland; Institute of Postgraduate Medical Education and Research, INDIA

## Abstract

**Background:**

Despite the overall decrease in visceral leishmaniasis (VL) incidence on the Indian subcontinent, there remain spatiotemporal clusters or ‘hotspots’ of new cases. The characteristics of these hotspots, underlying transmission dynamics, and their importance for shaping control strategies are not yet fully understood and are investigated in this study for a VL endemic area of ~100,000 inhabitants in Bihar, India between 2007–2015.

**Methodology/Principal findings:**

VL incidence (cases/10,000/year) dropped from 12.3 in 2007 to 0.9 in 2015, which is just below the World Health Organizations’ threshold for elimination as a public health problem. Clustering of VL was assessed between subvillages (hamlets), using multiple geospatial and (spatio)temporal autocorrelation and hotspot analyses. One to three hotspots were identified each year, often persisting for 1–5 successive years with a modal radius of ~500m. The relative risk of having VL was 5–86 times higher for inhabitants of hotspots, compared to those living outside hotspots. Hotspots harbour significantly more households from the two lowest asset quintiles (as proxy for socio-economic status). Overall, children and young adelescents (5–14 years) have the highest risk for VL, but within hotspots and at the start of outbreaks, older age groups (35+ years) show a comparable high risk.

**Conclusions/Significance:**

This study demonstrates significant spatiotemporal heterogeneity in VL incidence at subdistrict level. The association between poverty and hotspots confirms that VL is a disease of ‘the poorest of the poor’ and age patterns suggest a potential role of waning immunity as underlying driver of hotspots. The recommended insecticide spraying radius of 500m around detected VL cases corresponds to the modal hotspot radius found in this study. Additional data on immunity and asymptomatic infection, and the development of spatiotemporally explicit transmission models that simulate hotspot dynamics and predict the impact of interventions at the smaller geographical scale will be crucial tools in sustaining elimination.

## Author summary

Visceral leishmaniasis (VL) is the deadliest vector-borne parasitic disease after malaria worldwide and is one of the neglected tropical diseases targeted for elimination and control by the World Health Organization. Despite the overall decrease in VL incidence in Bihar, India, a region previously highly endemic for VL, there remain hotspots of cases for reasons that are poorly understood. This study demonstrates clear spatiotemporal heterogeneity of VL in an area of ~100,000 population in Muzaffarpur, Bihar, India between 2007–2015. Identified hotspots occurred almost always in the poorest communities, confirming that VL is a disease of ‘the poorest of the poor’. Our finding that older age groups (35+ years) are at a comparable high risk for VL within hotspots, similar to the 5–14 years highest risk group, but not outside hotspots, suggests a potential role of waning immunity as driver of hotspots. To further understand the drivers and transmission dynamics underlying VL hotspots, and ultimately inform policy, additional longitudinal data are needed to understand more about the role of asymptomatic infections, human movement, sand fly distribution and immunity. Including hotspots in mathematical transmission models is relevant for predicting the feasibility of reaching and sustaining VL elimination at a smaller geographical scale.

## Introduction

Visceral leishmaniasis (VL)–also known as kala-azar—is the deadliest vector-borne parasitic disease after malaria worldwide and is transmitted by female sand flies [1–3]. On the Indian subcontinent (ISC), VL is caused by *Leishmania donovani* and humans are considered the only host. Here, most of the infected individuals remain asymptomatic carriers of the parasite [4]. When symptoms do develop, they include prolonged fever, enlarged spleen and liver, anaemia and anorexia, leading to death when left untreated [5]. Globally, an estimated 15,000 to 65,000 cases occur each year [3,6,7]. VL is one of the neglected tropical diseases that has been targeted for elimination and control by the World Health Organization (WHO) [8]. For VL, the elimination target is defined as an annual VL incidence of <1 case per 10,000 capita at (sub)district level by 2020 on the ISC [8–10]. In the rest of the world, where the disease is also zoonotic, the WHO target for VL control is 100% detection and treatment of VL cases [11]. It has been estimated that the global health and economic gains from reaching these WHO targets would be enormous [12]. Interventions against VL on the ISC are based on early detection and treatment of cases and vector control, mainly through indoor-residual spraying of insecticide (IRS) [5,11]. In 2005, the governments of India, Nepal and Bangladesh committed to large-scale VL elimination programs [13]. Over the past decade, VL incidence rates have decreased at subcontinent, country and regional levels, which may be attributable to these efforts or to overall socio-economic improvements [14–18]. Bihar state in northern India is the area most affected by VL, and accounts for approximately 80% of all reported VL cases on the ISC. Despite the overall decrease in VL incidence in Bihar, which in many places has reached or is nearing the elimination target, there remain hotspots of infection [19–24]. The drivers underlying VL hotspots and the duration and magnitude of the flare-ups at small-scales are not yet fully understood.

In the past seven years different mathematical models that capture the transmission dynamics of VL, have been developed by Stauch *et al*. [13,25] and research groups of the NTD Modelling Consortium (http://www.ntdmodelling.org/) to better understand VL transmission dynamics and to predict the impact of control, in particular the feasibility of achieving the WHO elimination target for the ISC with current control strategies [11,13,24–27]. All models have been based on data from VL endemic areas in India, Nepal and Bangladesh [11,26], and currently do not include any spatiotemporal dynamics, besides the seasonal sand fly patterns. These models assume that without interventions, an endemic equilibrium is reached with homogenous distribution of cases, and currently do not simulate the presence of hotspots.

The aim of this study is to gain insight into the spatiotemporal patterns and hotspots of VL incidence at hamlet level (subunit of a village) and their underlying drivers—using longitudinal case and population data from a study area in Muzaffarpur, Bihar, India [22,28]. At the local level, VL dynamics are known to be influenced by socio-economic status and immunity among other factors [1,28,29]. This has been confirmed for the study area during previous analyses, where belonging to the wealthiest quintile has proven to be protective. Inhabitants from this quintile have an odds ratio (OR) of 0.5 (95% confidence interval (CI) 0.3; 1.0) of acquiring VL, compared to the poorest asset quintile [1,28]. We hypothesise that—even in this era of decreasing VL incidence—VL outbreaks continue to arise in areas where relatively many people from the lower asset quintiles live. In addition, children and young adolescents (5–14 years) are known to have the highest risk of developing clinical VL compared to the (0–4 years) reference category, due to either more exposure to the parasite or a lack of immunity (OR 2.5, 95% CI 1.5; 4.0) [28]. According to previous studies, long-lasting immunity might be acquired after exposure to the parasite [11,30]. It is hypothesized that all people living in VL endemic areas like rural Muzaffarpur, at least in pre-control settings, are exposed to *L*. *donovani* at some point in their lives. Most people remain asymptomatic, just a small fraction of people develop clinical symptoms [4]. It is assumed that hamlets and villages get saturated over time and only susceptible inhabitants cause new flare-ups or ‘hotspots’ due to new-borns, migration, or waning immunity of those with past (asymptomatic) infection [25,29]. Both factors, poverty and age, are analysed in this study as potential drivers of hotspots.

A better understanding about the presence of spatiotemporal clustering of VL at hamlet level and the underlying characteristics of such hotspots can aid in further shaping the intervention strategies towards 2020 and can be used to inform mathematical transmission models, subsequently improving their predictions regarding the feasibility of achieving and sustaining the VL elimination targets at a smaller geographical scale.

## Methods

### Data

**Fig 1:**
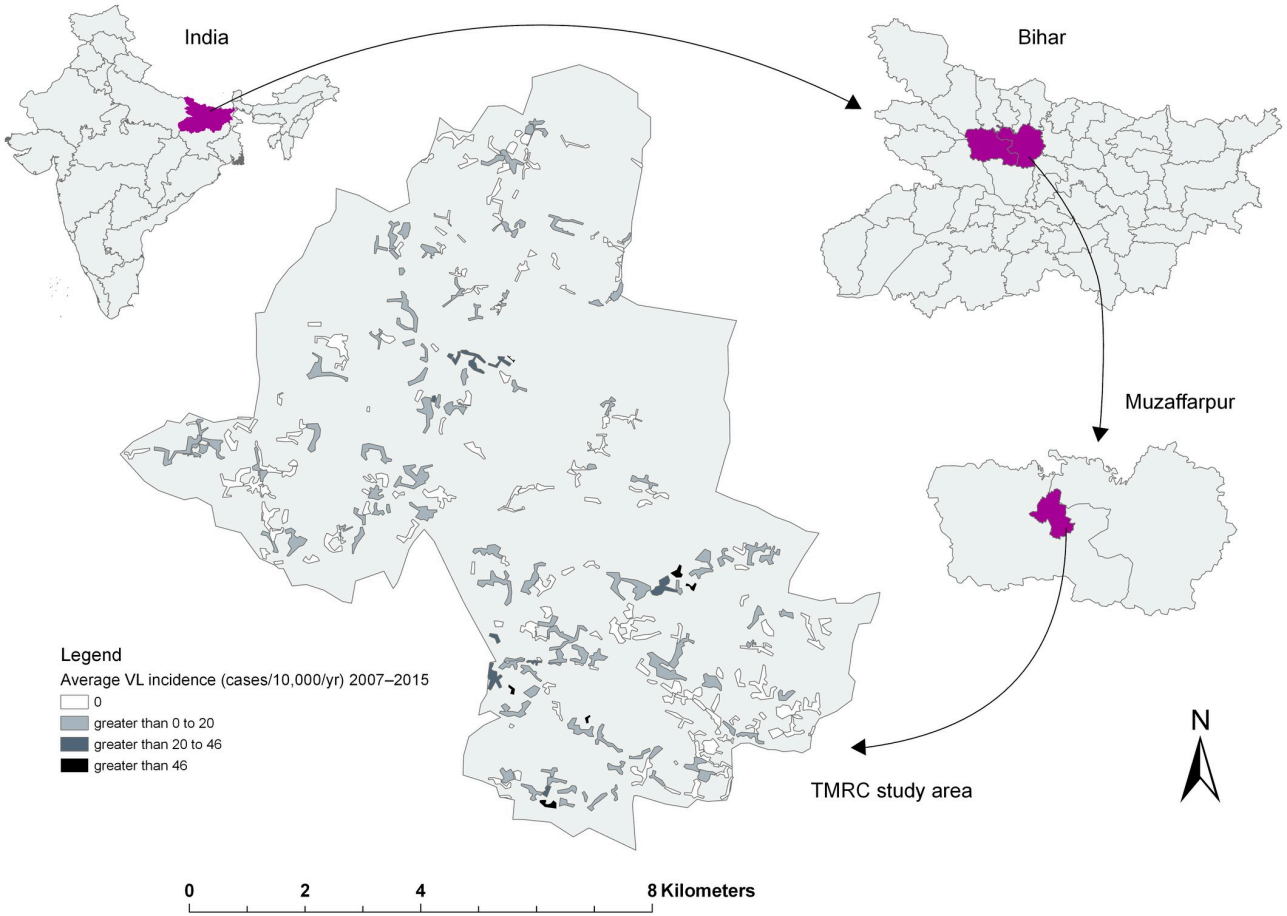
Location map of the study area, presenting the average number of visceral leishmaniasis (VL) cases per 10,000 capita per year for each hamlet (subunit of a village) between 2007 and 2015.

We exploited the georeferenced data on VL cases as well as demographic data on all households obtained in the “Muzaffarpur—TMRC Health and Demographic Surveillance System” for the period between 2007 and 2015 [31]. This study site is located in a densely populated rural area of the Kanti block (or subdistrict) in Muzaffarpur district, Bihar state, India (Fig 1), a region marked as a hyper-endemic for VL [22,28]. The study area covers approximately 85 km^2^ lying between 26.13054°N– 26.23554°N latitude and 85.16642°E– 85.27294°E longitude and includes 2% of the total population of Muzaffarpur district (Census 2011). The study area comprises 50 villages that can be further subdivided into 276 hamlets, also known as *tola*. Almost 100,000 inhabitants from ~13,500 households agreed to be enrolled in the study (90% of all households in the area) and were followed annually. Cases were identified through active door-to-door surveys and a VL case was defined as “the combination of a clinical history typical for VL (fever of >2 weeks’ duration with lack of response to antimalarial drug treatment); a positive result in the rK39 rapid diagnostic test; and a good response to VL treatment” [28]. The documented year of detection of the case was based on the date of diagnosis and onset of treatment. Indoor residual spraying of houses in the region started in 2005 and has been ongoing ever since, but coverage has been poor [28]. Muzaffarpur has a humid tropical climate with temperatures ranging from 18.5 to 31.9 °C and an average rainfall around 1,300 mm annually. Bihar belongs to the five poorest states in India, and the inhabitants of Muzaffarpur district belong to the low to middle income segments of the Bihar wealth distribution [1,28,32].

The asset index of every household and the age of all inhabitants were assessed at the start of the study and were available for all VL cases detected between 2007 and 2015. The asset index was based on the household wealth and living standards of Bihar and was determined by the type of housing and assets owned by the household like tables, chairs, bicycles and motorcycles. The asset index was assumed to remain stable throughout the study [1,28]. The variable “asset index” was transformed into quintiles (with 20% of individuals belonging to category ‘1’ the poorest quintile of the Kanti block study population to 20% in ‘5’ the wealthiest population quintile within the area, measured at household level). An overview of the asset quintile distributions with the exact calculations is provided by Boelaert *et al* (2009) [1].

### Visualisation of VL incidence trends and spatiotemporal heterogeneity

We calculated and plotted the overall annual VL incidence rates per 10,000 capita for each year of the study period and annual incidence rates for each hamlet. The annual incidence rates of hamlets with non-zero case counts are shown in a histogram. The geospatial heterogeneity in annual VL incidence was visualized with ArcGIS Pro version 2.1.0 (www.esri.com). Hamlets were grouped into four incidence categories using the same cut-off values for all nine years. All hamlets with an annual VL incidence of zero VL cases per 10,000 capita were grouped into the same category. Based on the histogram, the remaining three categories were as follows: greater than 0 to 20, greater than 20 to 46 and greater than 46 VL cases per 10,000 capita per year.

### Temporal and spatial autocorrelation

As with other infectious diseases, chances that high VL incidence rates are present within a hamlet in one year, are expected to be higher for hamlets with high VL incidence rates in the previous year, compared to hamlets that had no cases in the previous year [33,34]. Patterns and autocorrelation over time in VL incidence within hamlets were quantified with generalized linear mixed models. We compared the predictive performance of negative binomial and Poisson models with or witout random intercepts and slopes over time using k-fold cross valiation (k = 10). In addition to random intercepts and slopes we tested if adding a gaussian process for temporal autocorrelation (periods of consequtive years with outbreaks) improved the models’ predictiove performance. Models were fitted using the rstan and brms packages in R version 3.3.3.

Spatial autocorrelation or clustering in interpolated annual VL incidence was assessed using Moran’s *I* index [35]. The *I*-statistic produces values range from -1 to +1. An ‘*I*’ value around 0 suggests features with similar values are randomly distributed in space. An ‘*I*’ value significantly different from 0 suggests a pattern. Values close to +1 suggest positive autocorrelation, whereas a value close to -1 suggests negative autocorrelation. Significant (p<0.05) positive autocorrelation indicates that features (hamlets in this study) are surrounded by features with similar values, which means the feature is part of a cluster [23,36].

### Hotspot detection analyses

We used a Poisson model through Kulldorff spatial scan statistics (www.satscan.org) to detect spatial clusters of high VL incidence or ‘hotspots’ for each year and spatiotemporal hotspots for the overall study period (2007–2015). The model provides a series of Poisson-based draws against which annual VL incidence rates are compared. Under the null hypothesis, stating that cases are randomly dispersed in space, the expected number of cases is proportional to the population size of the area [37]. The analysis requires case and population counts for a set of data locations, as well as the geographical coordinates for each of the locations. We constructed a model with the following conditions: searching for the most likely hotspots without geographical overlap of hotspots within the same time frame and maximum radius of a cluster equal to average maximum semivariogram range. The semivariogram range represents a distance beyond which there is little or no spatial autocorrelation among variables and was measured by fitting semivariograms of the incidence data at hamlet level per 3-year time frame (2007–2009, 2010–2012 and 2013–2015). For each detected hotspot, the number of hamlets within the hotspot, radius (km), population at risk within the hotspot and the observed number of cases versus the expected number of cases detected within the hotspot are given. The risk of having VL that is associated with an identified hotspot is presented as the relative risk (RR), i.e. the ratio of estimated VL risk inside relative to outside a hotspot. Only significant (p<0.05) hotspots were included in our further analyses.

Incidence (cases/10,000/year) of each significant spatiotemporal hotspot were plotted over time to quantify the duration of VL outbreaks, hotspots were defined as ‘outbreaks’ when the incidence within a hotspot rose above 15 cases per 10,000 per year.

### Potential drivers underlying VL hotspots

Socio-economic status and age were analysed as potential drivers of VL hotspots, using multiple approaches. Two different methodologies were used to investigate a potential association between VL hotspots and socio-economic status (measured by asset index). First, we compared the fraction of households in the different asset index quintiles of the study population, inside and outside of annual hotspots, using a Chi-squared test. A p-value of <0.01 was taken as the level of significance because of the large sample size of almost 100,000 inhabitants from ~13,500 households. We calculated the percentage difference and the 95% CI around the perectage difference of households within the poorest two asset quintiles inside and outside of hotspots, and the fractions were considered significantly different if zero did not fall into the CIs. Second, we investigated the potential association between VL incidence and poverty at the hamlet level by means of Poisson regression, with poverty being defined as the percentage of households within the two poorest asset quintiles of a hamlet.

VL immunity is known to be linked to age and we used different methodologies to study the association between VL and age in relation to detected hotspots. First, we calculated the age distribution of inhabitants and VL cases living inside and outside of hotspots. We compared the age distribution of VL cases (fraction 5–14 years versus all other ages) using a Chi-squared test, a p-value of <0.05 was taken as the level of significance, because of the relatively small sample size of 329 VL cases. The age distribution of VL cases living within hotspots that were identified for the overall study period was investigated, by looking at the time-frame of outbreaks and splitting the outbreak into a first phase (up to the peak) and a later phase. A Chi-squared test was used to test if the age distributions (5–14 years versus the rest) were significantly different when comparing the early with the later stage of an outbreak.

Further, a binomial logistic regression model was created with age (0–4, 5–14, …, 35–44 and 45+ years) and asset index as categorical predictors and hamlet as a random effect. The groups known to have the highest odds for VL in rural Muzaffarpur [28], age 5–14 years and asset quintile 1, were used as the reference categories for calculating the adjusted odds ratios. Whether an individual lived inside a detected hotspot or not, was added as a dichotomous predictor (inside *vs*. outside), allowing for potential interaction with age and asset index. The Akaike Information Criterion (AIC) was used to determine whether adding of predictors and interactions between predictors improved the model fit.

The difference in age distribution of VL cases over time was assessed by splitting outbreaks into an upward phase (up to the peak) and downward phase.

## Results

### Annual VL incidence trends and heterogeneity

**Fig 2:**
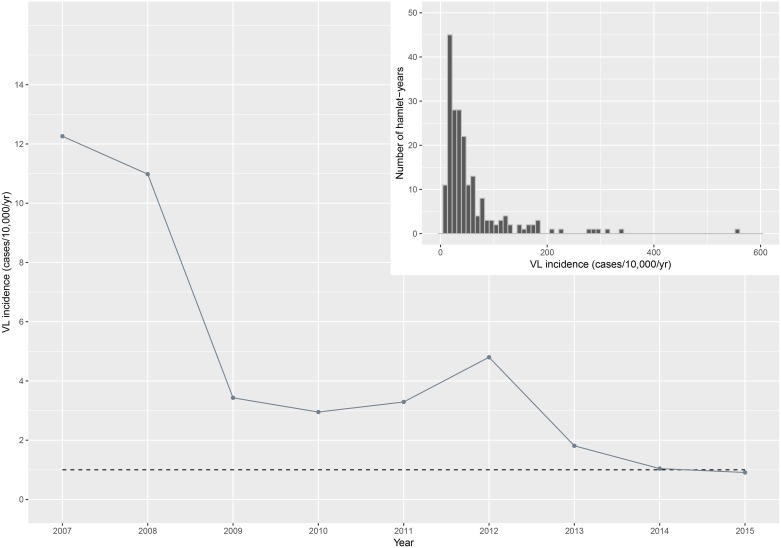
Incidence (cases/10,000/year) of visceral leishmaniasis (VL) in the study area between 2007 and 2015. The dashed line represents the WHO 2020 elimination target [10]. Top right panel: distribution of VL incidence (cases/10,000/year) at hamlet level shown for each year of the study period (*n* = 2,484), only the annual incidence rates of hamlet-years with case counts are shown in the histogram (*n* = 205).

A total of 329 VL cases were detected between 2007 and 2015, the average incidence was 4.2 cases per 10,000 per year within the study area. The highest annual VL incidence of 12.3 cases per 10,000 per year was documented in 2007 (when the study started), followed by a steady decrease up to 2010. In 2012 a small peak was observed. Annual incidence rates further dropped in later years of the study period and reached the elimination threshold in 2015, when the annual VL incidence was 0.9 cases per 10,000 per year. Overall annual VL incidence rates between 2007 and 2015 and the 2020 elimination target are illustrated in Fig 2. Annual VL incidence rates vary highly across the hamlets (mean incidence 4.7 cases per 10,000 per year, range 0.0–555.6 cases per 10,000 per year). Around 40% of all hamlets (114 out of 276) captured all reported VL cases between 2007 and 2015. Of all 2,484 hamlet-years (276 hamlets times nine years), there were 205 hamlet-years with VL case counts, and 57% of these hamlet-years with case counts occurred during the first three years of the study (2007–2009). Almost half (48%) of these hamlet-years had at least one neighouring hamlet (within a range of 500 meters) with reported VL cases during the same year. Furthermore, 65% of hamlets with reported VL cas es arose in the periphery around hamlets with reported VL cases in the previous year; 59% in or around hamlets with reported VL cases two years from that year and 53% in or around hamlets with reported VL cases three years from that year. The histogram (top right panel of Fig 2) shows the incidence (cases/10,000/year) at hamlet level for all hamlet-years with VL case counts in that year. The median age of cases was 20 years, the youngest detected case was 2 years old and the oldest was 70 years.

### VL temporal autocorrelation and hotspots over time

Generalized linear mixed effects regression for VL incidence over time within a hamlet revealed that VL incidence rates are temporally clustered for 1.6 years (95%-BCI 1.046–2.555). The full model output is provided in S1 Table.

### Spatial autocorrelation and geographical scale of VL hotspots

**Fig 3:**
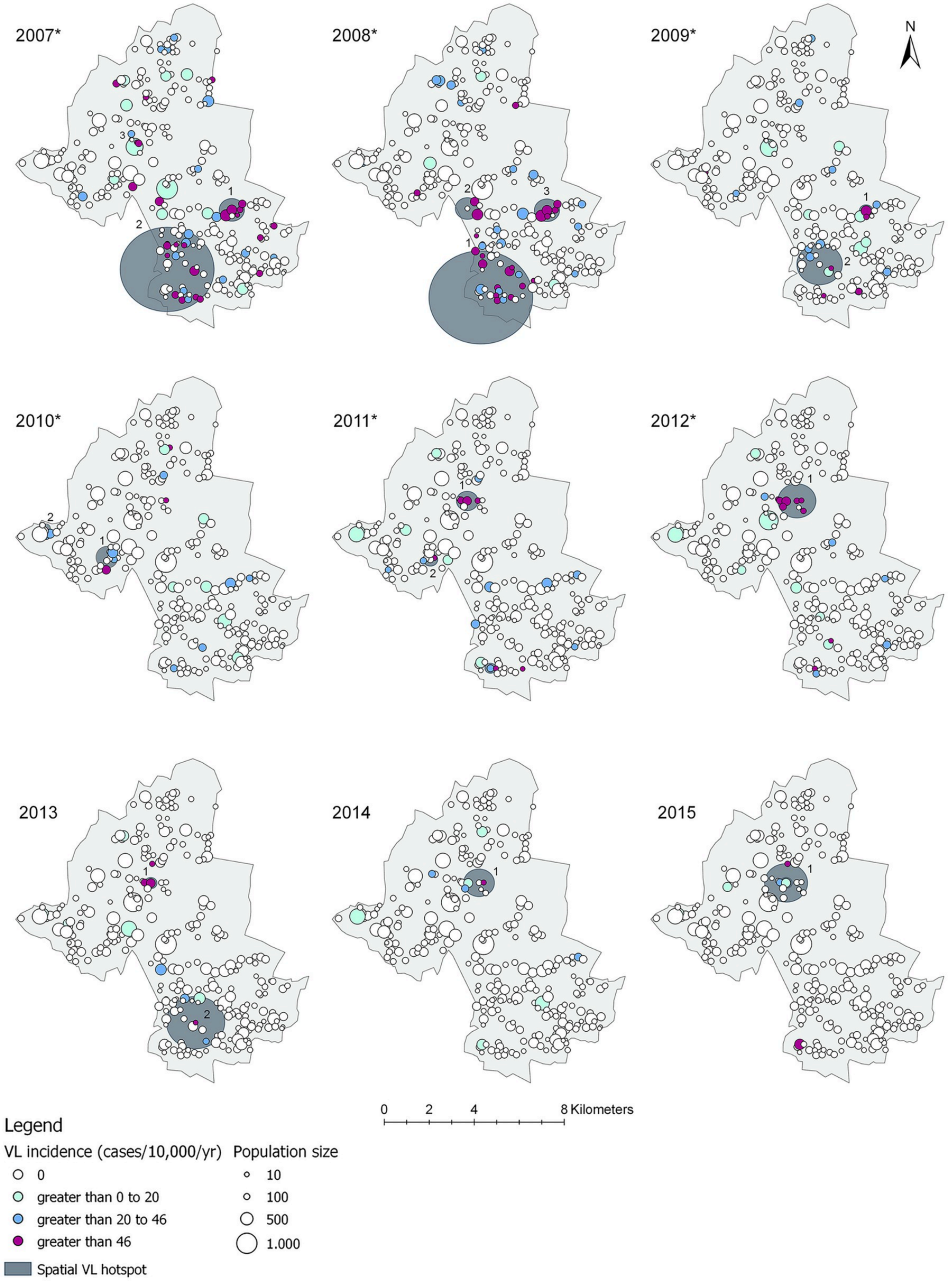
Spatial distribution and incidence (cases/10,000/year) of visceral leishmaniasis (VL) per hamlet, with detected hotspots (dark grey circles) within the study area of Muzaffarpur, Bihar, India between 2007 and 2015. The years with an Asterix (*) represent significant spatial autocorrelation of VL incidence between hamlets as identified using Moran’s *I*. The cluster numbers correspond with the numbers of the clusters identified per year using spatial scan statistics (S3 Table).

Mapping the geographical distribution of VL illustrates the spatial heterogeneity in endemicity among hamlets in rural Muzaffarpur (Fig 3). Moran’s *I* index (S2 Table) showed that VL incidence rates per 10,000 per year were significantly spatially clustered at hamlet level in 2007 to 2012. P-values below <0.05 or <0.01 suggest that the likelihood of this spatial pattern being random, i.e. a result of chance, is less than 5 or 1 percent respectively. In 2013–2015, Moran’s *I* values approach zero and were not statistically significant, suggesting that no spatial pattern could be identified among hamlets with low and high incidence rates per 10,000 capita during these years. The combined semivariogram of the incidence data at hamlet level (S1 Fig) demonstrated that the maximum distance beyond which there is little or no autocorrelation among hamlets over 2 km. One to three significant spatial hotspots of VL were detected in each year. The hotspots include two to 40 hamlets and have a radius between 85 meters and 1.9 km (modal radius of 500 meters). The largest hotspot covers a surface of 11 km^2^. A total of 82 out of 276 hamlets were located within a hotspot during at least one year. The relative risk of having VL associated to living inside a hotspot is 5 to 86 higher relative to outside a hotspot. A full overview of the hotspots detected with spatial scan statistics is displayed in S2 Table.

**Fig 4:**
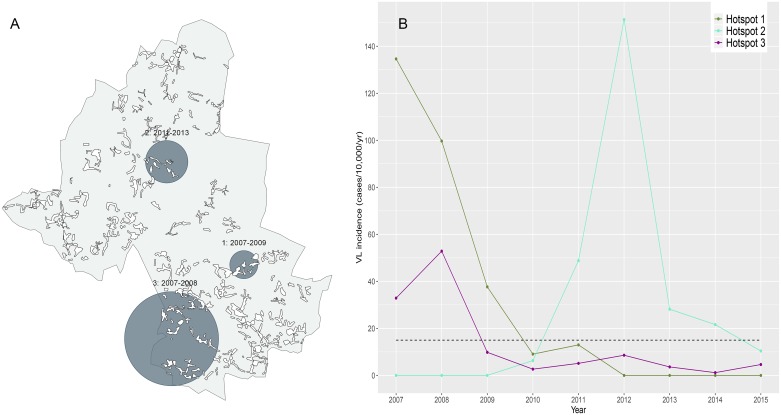
Spatiotemporal hotspots of visceral leishmaniasis (VL) within the study area in Muzaffarpur, Bihar, India shown spatially (dark grey circles, panel A) and over time (panel B). The black dashed line in panel B represents the cut-off value for an ‘outbreak’, where at least three annual cases are detected within a hotspot (VL incidence of ≥15 cases per 10,000 per year).

Three significant spatiotemporal hotspots that were detected over the overall study period (Fig 4). The hotspots have a duration of two to three years (Fig 4, panel B). A detailed overview of the characteristics of the spatial and spatiotemporal hotspots is given in S3 Table.

### Potential drivers of VL hotspots

**Fig 5:**
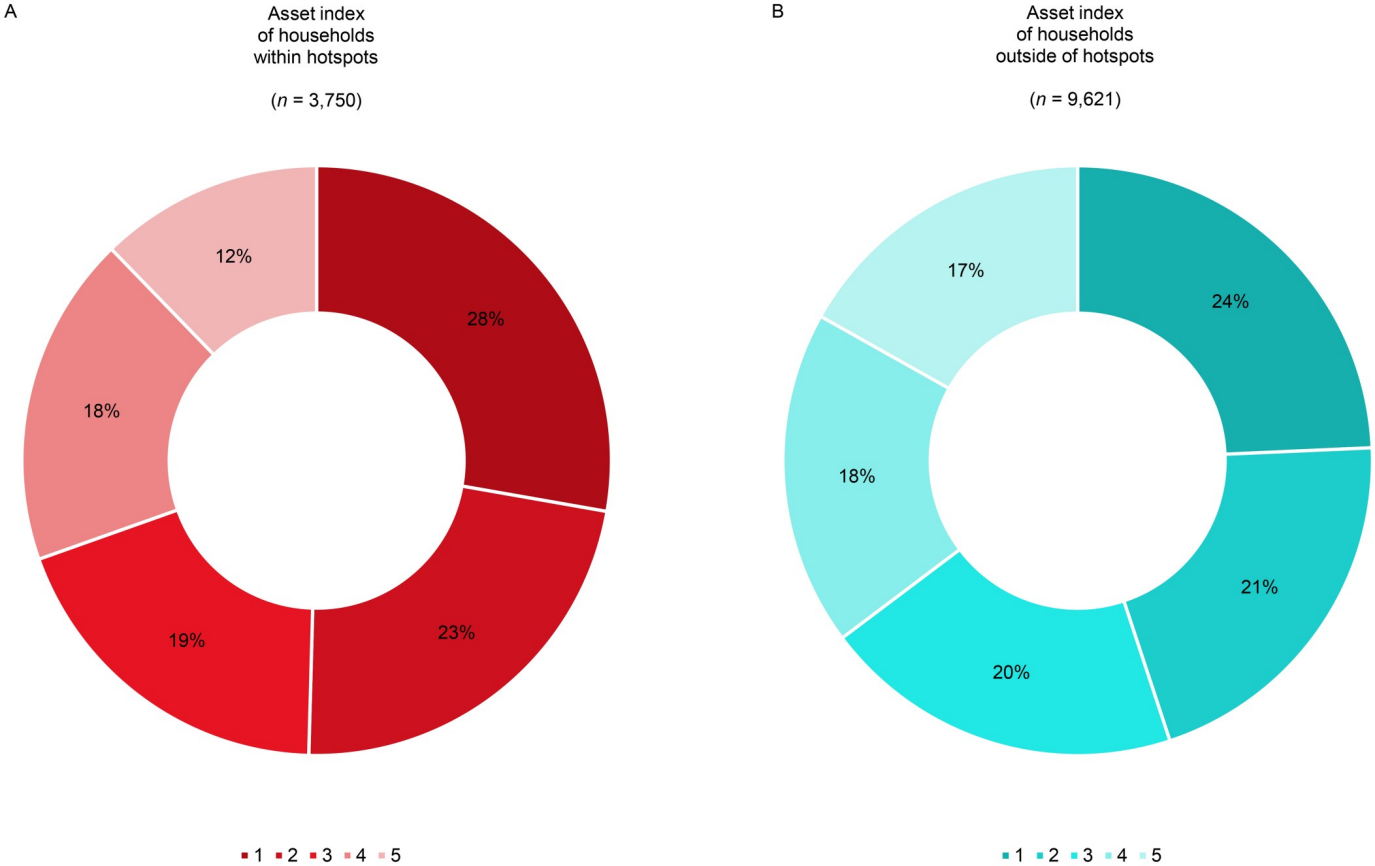
Asset index distribution (‘1’ poorest to ‘5’ wealthiest) of households located within annual hotspots during at least one year (panel A) and outside of the hotspots (panel B), where ‘*n*’ is the total number of households.

**Fig 6:**
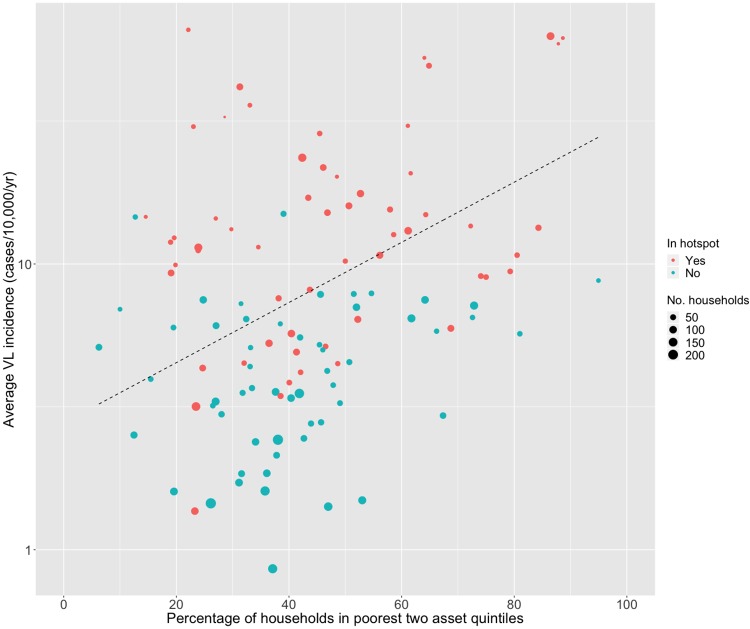
Visceral leishmaniasis (VL) incidence and poverty at the hamlet level. The black dashed line illustrates the Poisson regression line, where 10% more households within the two poorest asset quintiles in a hamlet come with 2.4% increase in annual incidence.

Fig 5 shows the asset index distribution among households located inside (panel A) and outside hotspots (panel B). This distribution differed significantly between households inside and outside hotspots ($\chi^{2}$ = 54.7, degrees of freedom (df) = 2, p<0.001), with hotspots holding 5% (95% CI 4.94%; 5.06%) fewer wealthy (12% *vs*. 17%) and 6% (95% CI 3.31%; 8.69%) more poor households (51% *vs*. 45%). VL incidence and poverty at the hamlet level are significantly associated (p<0.001): hamlets with a higher VL incidence harbour relatively more households from the two poorest asset quintiles (Fig 6 and Box 1).

**Box 1. What are potential drivers underlying visceral leishmaniasis (VL) hotspots at hamlet (subvillage) level?**

- Poverty—The poorest of the poor populations have an increased VL risk [1,28], and our study shows that within VL hotspots, significantly more households belong to the two poorest asset quintiles.
- Age and immunity—Children and young adolescents have an increased VL risk in the study area [28], possibly linked to a lack of immunity. If outbreaks are also driven by waning herd immunity, we would expect cases within hotspots to be older compared to cases outside of hotspots [44]. Our study shows that older age groups (35+ years) have a comparable high risk of having VL within hotspots relative to the 5–14 years high-risk group, whereas their risk was significantly lower outside of hotspots. There also is a shift towards younger ages, among cases reported during the downward phase of VL outbreaks. Both findings suggest a role of waning herd immunity.

**Fig 7:**
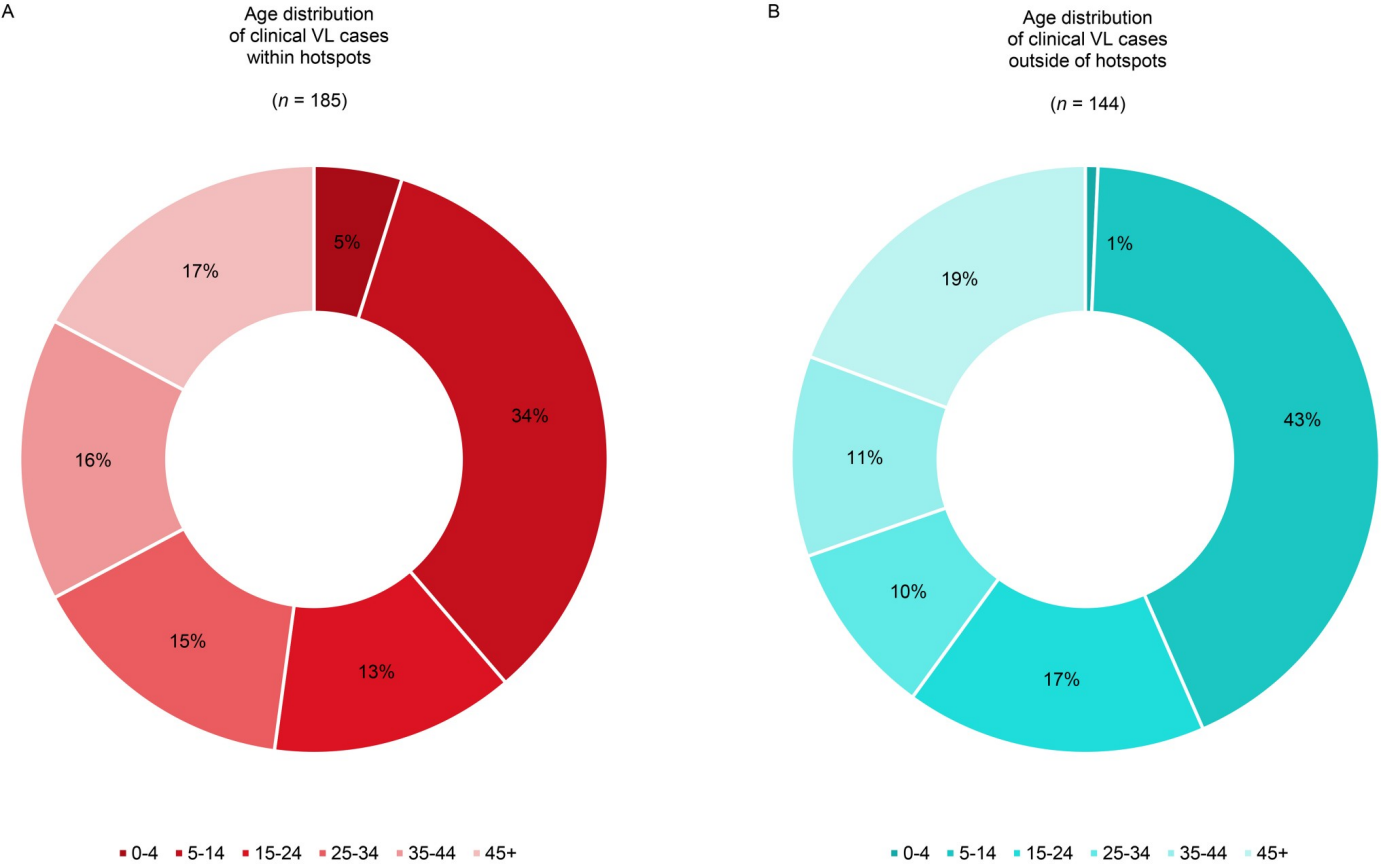
Age distribution of all visceral leishmaniasis (VL) cases detected between 2007 and 2015 living within (panel A) and outside hotspots (panel B) at time of detection, where ‘*n*’ is the total number of cases detected.

The age distributions among VL cases that were detected within and outside of the hotspots are illustrated in Fig 7. In the VL hotspots, 9% (95% CI -0.08%; 0.26%) less cases were between 4 and 15 years old (34% *vs*. 43%), whereas the fraction of the entire population in the same age group was very similar inside and outside hotpots (27% *vs*. 26%, S3 Fig). The fraction of all VL cases belonging to the 5–14 years age group is not significantly different inside relative to outside of hotspots ($\chi^{2}$ = 2.4, df = 1, p-value = 0.123). S4 Fig shows there is no significant association between the average age of VL cases and annual incidence at the hamlet level for both hamlets within hotspots and outside of hotspots.

The results presented in Table 1 illustrate that outside of hotspots, the risk of having VL is lower for all age groups compared to the reference group (5–14 years), whereas inside of hotspots, the older age groups (35–44 and 45+ years) have a higher risk for VL compared to the reference group, also after adjusting for asset index. Comparable to the findings by Hasker *et al*. [28], people from the highest asset quintile had the lowest risk of having VL, compared to the poorest asset reference group (Box 1). However, there was no difference in the association between asset index distribution and VL when comparing the people living inside to those outside of hotspots; adding an interaction term between asset index and hotspot did not improve the model fit (AIC = 3794.6 *vs*. 3789.9).

**Table 1:** Logistic regression showing the association between visceral leishmaniasis (VL) status, age and asset index (as proxy for socio-economic status) for hotspots and areas outside of the hotspots.

Covariate	Cases per 10,000 capita^a^	Number of cases^a^	Population size	OR (95% CI)	P-value
**Total**	40.5	329	81,214	-	-
***Location***					
** Outside a hotspot**	24.4	144	58,961	1	-
** Inside a hotspot**	83.1	185	22,253	4.48 (2.82; 7.10)	<0.001 ***
***Age group outside of hotspots***					
** 0–4**	1.1	1	9,300	0.04 (0.01; 0.27)	0.001 ***
** 5–14**	41.1	62	15,090	1	-
** 15–24**	23.2	24	10,336	0.72 (0.42; 1.22)	0.218
** 25–34**	17.5	14	7,997	0.41 (0.20; 0.85)	0.016 *
** 35–44**	24.3	15	6,185	0.58 (0.29; 1.16)	0.123
** 45+**	27.9	28	10,053	0.44 (0.23; 0.83)	0.012 * ^b^
***Age group inside of hotspots***					
** 0–4**	25.8	9	3,487	0.16 (0.08; 0.33)	<0.001 ***
** 5–14**	106.2	63	5,931	1	-
** 15–24**	63.3	25	3,947	0.57 (0.37; 0.88)	0.011 *
** 25–34**	93.5	28	2,996	0.81 (0.54; 1.23)	0.327
** 35–44**	123.0	28	2,277	1.18 (0.79; 1.77)	0.412
** 45+**	88.5	32	3,615	1.07 (0.74; 1.54)	0.715 ^b^
***Asset index***					
** 1 ‘Poorest’**	63.6	105	16,511	1	-
** 2**	57.4	92	16,036	0.90 (0.67; 1.20)	0.456
** 3**	35.8	58	16,191	0.63 (0.45; 0.88)	0.007 **
** 4**	30.8	50	16,249	0.56 (0.39; 0.79)	0.001 **
** 5 ‘Wealthiest’**	14.8	24	16,227	0.32 (0.20; 0.51)	<0.001 ***

* = p<0.05;

** = p<0.01;

*** = p<0.001

^a^ = Based on the cumulative number of VL cases over the 9-year study period.

^b^ = Significant p-value (p<0.05) for the interaction between age and hotspot, i.e. people had a significantly higher risk of having VL (relative to the 5–14 years old reference group) when 45+ years inside a hotspot, whereas this was not the case outside a hotspot.

We further investigated the potential role of progressively developing herd immunity at this small area level by comparing the age distribution of VL cases between the upward and the downward trend of small-scale VL outbreaks. For this, we used hotspots 1 and 2 (Fig 4) as these hotspots showed clear peaks in VL incidence (≥15 cases/10,000/year) and may therefore be considered as local outbreaks. Among the VL cases detected in the downward phase of a local ‘outbreak’ (hotspot 1 and 2 combined), there was a shift towards younger ages, compared to cases detected during the upward phase of an outbreak (hotspot 2) (S5 Fig).

## Discussion

This data collected over a 9-year period in the heartland of the VL epidemic in India provided a unique opportunity to analyse changing patterns in VL epidemiology. The study demonstrates significant spatiotemporal heterogeneity in VL incidence at the subdistrict level in Bihar, India, and is the first study to investigate the drivers underlying hotspots at this geographical scale.

The overall decrease in VL incidence observed in the area is comparable to the steep case declines observed in many other VL endemic areas on the ISC during the past ten years [17,38]. Despite the overall decrease, hotspots were identified during each year of the study period. The geospatial and (spatio)temporal clustering patterns found in this study (Box 2) are comparable to the findings from other recent studies conducted in India and Bangladesh [39–41]. A recent study by Mandal *et al*. (2017) discovered that 93% of newly endemic villages in the Indian Vaishali district, a district located at the border of Muzaffarpur, occurred on the peripheries of previous year endemic villages, suggesting clustering in both space an time. We found similar patterns in Muzaffarpur: 65% of the hamlets with VL cases arised in the peripheries (within a range of 500 meters) of hamlets with VL in the previous year [40]. The researchers did not use statistical alayses to further explore the observed VL patterns. Earlier, spatial clustering of VL incidence was identified in Vaishali by Bhunia *et al*. (2013). However, they used aggregated subdistrict level data to identify clustering, which differs from our geocoordinate based subvillage level approach. Moreover, only Moran’s *I* index and Getis-Ord Gi were applied. Both analyses separate clusters of high-high and low-low incidence levels, but do not specifically detect hotspots, like spatial scan statistics allows for [39]. Dewan *et al*. (2017) identified VL hotspots using the same analysis methods (Moran’s *I* followed by Poisson spatial scan statistics) at the *mauza* (cluster of several villages) level in an endemic area of Bangladesh [41]. All four identified hotspots had a ~2 km radius and inhabitants of hotspots had a 4 to 11 times higher relative risk of having VL, compared to people living outside of hotspots. In our study, hotspots had a 85 meters to 2 km radius and inhabitants of hotspots had a 5 to 86 times higher relative risk of having VL. So, at this smaller geographical scale, hotspots are smaller and have a higher relative risk, compared to the findings by Dewan *et al*. (2017). These findings might suggest that the smaller the geographical scale of analysis, the stronger clustering of VL is present.

**Box 2. Epidemiologic insights: What do identified spatiotemporal patterns of visceral leishmaniasis (VL) tell us about the scale of transmission?**

- Around 40% of hamlets capture all VL cases in the study area of approximately 85 km^2^ and ~100,000 population over the 9-year study period.
- Temporal clustering of low-low and high-high VL incidence levels within a hamlet persisted for 1.6 years and 65% of the hamlets with reported VL cases had VL cases or neighnouring hamlets with VL cases (within a 500-meter radius) in the previous year. One to three significant hotspots were identified each year, often persisting for 1–5 successive years.
- Spatial and spatiotemporal hotspots in the study area had a radius of 85 meters to around 2 km (modal radius 500 meters), covering areas up to 11 km^2^.
- While VL incidence rates varied from 0.9 to 12.3 cases per 10,000 per year for the total study area, incidence rates in hotspots ranged from ~13 to ~300 cases per 10,000 per year.

Within hotspots, the relative risk of having VL was 5 to 86 times higher than outside of hotspots.

Our findings suggest that hotspots are, at least partially, driven by poverty, since hotspots arise more frequently in hamlets with the highest proportion of ‘poorest of the poor’. We hypothesised that hotspots might develop in areas with a relatively large susceptible population, leading to relatively older VL cases within hotspots, most clearly during the early stage of outbreaks. Our finding that outside hotspots the risk of VL was significantly lower among people of age 45+ years of age compared to people of age 5–14 years, but was comparable between these two age categories inside hotspots, suggests a potential role of long-lasting immunity as an underlying driver of heterogeneity in VL incidence. However, additional data on immune status are needed to further explore this hypothesis. The steep decrease in overall VL incidence rates observed over the 9-year study period may be the result of intervention strategies in place—even though IRS was mentioned to be suboptimal within the study area—making it challenging to interpret the temporal trends in VL incidence [28]. Furthermore, possible natural cycles or development of herd immunity might have played a role in the observed downward trend [20,42–44]. In contrast to the research by Hasker *et al*. [26], other studies pointed out that being 15 years or older came with an increased risk of developing VL [45,46]. Outbreaks at this small geographical scale might be explained by specific risk factors increasing susceptibility to *L*. *donovani* infection, like migration and immunosuppression (mainly due to HIV-co-infection) [19,20,30,46]. The risk related to proximity of VL cases, could also be another underlying driver of development of VL, as was explored by Chapman *et al*. [47] and Hasker *et al*. [48]. The size of hotspots make it is most likely that both human movement and the movement of sand flies play an important role in the spread of infection [49–51]. The role of population density, access to health services, vector characteristics and vector control strategies, which have shown to be potent drivers of other vector-borne infections, would pose interesting additional factors to explore in future analyses as potential drivers of VL hotspots [23,52–56].

In current mathematical transmission models for VL, a homogeneous seasonal equilibrium of incidence is assumed at subdistrict level when no interventions are in place. Heterogeneity is included at individual level regarding age-dependent sand fly exposure [26]. To include spatiotemporal clustering of VL cases (hotspots) as an additional source of heterogeneity in these models, together with a human migration pattern, would represent a more realistic framework when simulating infection at the smaller geographical scale. This becomes especially relevant close to and in the first years after reaching the elimination target [11,25,26], in which case a stochastic individual-based transmission model would be a suitable tool for predictions. Since poverty partially explains the spatial heterogeneity—as families belonging to the same sociocultural group (caste) often live in close proximity—stratifying the population by socio-economic status in the current transmission models would probably better portray the spatial clustering of outbreaks in these populations. Current models assume that immunity lasts only for 1 or 2 years after infection (asymptomatic and/or symptomatic). Based on our findings, a longer immunity (i.e. ~15 to 30 years) would seem more realistic, and is also congruent with clinical observations, at least for clinical VL cases.

The identification of focal areas that are at greater risk for VL may help define priority areas of specific interventions when nearing the 2020 elimination target. Following our findings, active case detection might be focussed on areas where the ‘poorest of the poor’ are located when resources are limited. Based on our estimates, the current IRS policy of spraying all households within a radius of 500 meters from a detected case in less endemic areas, seems reasonable. However, the true impact of IRS on the number of VL cases remains uncertain [57]. Improving socio-economic conditions among the poorest of the poor households might be another effective control measure when targeting for VL elimination, but remains challenging.

Symptomatic VL cases are only ‘the tip of the iceberg’ and there are still many unknowns regarding the roles of asymptomatic individuals and people with post-kala azar-dermal leishmaniasis (PKDL) (Box 3). Previous studies have shown that “asymptomatic infection also tends to show clustering around VL cases” [29], and that having a new asymptomatic infection in a household puts the relatives at risk for developing VL or asymptomatic infection in the future [30]. Therefore, future research on *L*. *donovani* transmission in the post-elimination era should include asymptomatic individuals, by measuring Direct Agglutination Test (DAT) titres and rK39 antibody levels [58]. The ratio symptomatic: asymptomatic is estimated to be between 1:6 to 1:17 in India [4,59,60], this suggests that around the 329 clinical VL cases ~2,000 to ~6000 individuals were infected without showing any signs or symptoms. Though the ratio of incident asymptomatic infections to incident clinical cases seems to increase with decreasing transmission intensity [61], the overall prevalence of asymptomatic infections remains relatively low. Even when life-long immunity would be generated after an asymptomatic infection—what is not certain at this point—not all inhabitants of endemic hamlets will develop VL immunity during their lifetimes with the current incidence rates. The increasing pool of susceptible individuals that forms as VL incidence decreases may be a source of new large-scale epidemics of clinical cases—and this leads to the question whether elimination is desirable if one does not aim for the zero transmission goal [27].

**Box 3. What can we learn from this study for future visceral leishmaniasis (VL) data collection?**

- Clinical cases are only ‘the tip of the iceberg’. Future studies could focus on better understanding whether and how asymptomatic infections, human movement, sand fly distribution and waning herd immunity drive the existence of hotspots. This requires data on cellular immunity or cellular markers among populations located within and outside of VL hotspots.
- More longitudinal data, over longer timespans (>15 years), will be useful to advance our understanding of the natural cycles of VL.

Several study limitations could have affected our results. This study is, to our knowledge, the first study to assess temporal clustering of VL in endemic settings and to explore underlying drivers of spatial and spatiotemporal VL hotspots. Ideally, we would have used one model to simultaniously assess spatial and temporal autocorrelation. For example, by using a spatiotemporal model through Integrated Nested Laplace Approximation (INLA) [62]. However, these more advanced types of analyses are not easy to interpret and the applied methods in this study were sufficient in identifying spatial and temporal autocorrelation. Moreover, Muzaffarpur has always been one of the higher incidence districts, and it remains challenging to generalise our findings to low endemic settings. Heterogeneity is likely more evident in highly endemic settings, and these areas therefore best allow for investigating potential drivers of VL transmission dynamics. With deceasing overall VL incidence levels, larger study populations might be needed in the future.

In conclusion, spatiotemporal heterogeneity in VL incidence is evident at subdistrict level in Bihar, India. This heterogeneous and spatiotemporally clustered distribution of VL at hamlet level can be a useful feature to include in the next generation of mathematical transmission models. Hotspots are to some extent driven by poverty, illustrating VL as a disease of ‘the poorest of the poor’. The modal hotspot radius of 500 meters, and the average duration of hotspots of 1–5 years could be relevant for planning and targeting vector control and active case detection strategies. Data on asymptomatic infection, migration patterns, sand fly distribution are required to further understand the drivers and transmission dynamics underlying VL hotspots, as urgent questions need to be addressed with regard to the future investment in VL elimination. Should we try to sustain the current status of low transmission intensity or push transmission further down to zero?

## Supporting information

S1 Table Temporal autocorrelation in annual visceral leishmaniasis (VL) incidence rates at hamlet level between 2007 and 2015, showing that VL incidence is temporally clustered within a hamlet up to 1.6 years. (DOCX)

S2 Table Moran’s *I* index, estimating spatial autocorrelation in annual visceral leishmaniasis (VL) incidence rates at hamlet level between 2007 and 2015. (DOCX)

S3 Table Significant (p<0.05) spatial and spatiotemporal clusters of high visceral leishmaniasis (VL) incidence or ‘hotspots’ detected with Kulldorff spatial scan statistics for each year. (DOCX)

S1 Fig Semivariogram fitted on the annual incidence data at hamlet level for predicting the maximum distance beyond which there is little or no autocorrelation. (TIF)

S2 Fig Asset index distribution (‘1’ poorest to ‘5’ wealthiest) of visceral leishmaniasis (VL) cases detected between 2007 and 2015 living within hotspots (panel A) and outside of the hotspots (panel B), where ‘*n*’ is the total number of cases detected. (TIF)

S3 Fig Age distribution among the population located within annual visceral leishmaniasis (VL) hotspots (panel A) and outside of the hotspots (panel B), where ‘*n*’ is the total number of population. (TIF)

S4 Fig Association between the average age of visceral leishmaniasis (VL) cases and annual incidence at the hamlet level. The black dashed line shows the regression line representing the association between the average age of VL cases per hamlet per year and the annual VL incidence at hamlet level.
(TIF)

S5 Fig Age of reported visceral leishmaniasis (VL) cases during the upward phase (*n* = 33 cases) (panel A) and downward phase (*n* = 46 cases) (panel B) of an outbreak. The bars represent the percentage (on a log scale) of reported VL cases.
(TIF)
