# Supplementary material for: Visceral leishmaniasis: Spatiotemporal heterogeneity and drivers underlying the hotspots in Muzaffarpur, Bihar, India
Source: PLoS Negl Trop Dis. 2018 Dec 6;12(12):e0006888. doi: 10.1371/journal.pntd.0006888 (PMC6283467; doi:10.1371/journal.pntd.0006888)
Supplement: S1 Table — (DOCX) [file pntd.0006888.s001.docx]

|  | Posterior mean | Posterior standard deviation |
| --- | --- | --- |
| Intercept | -7.08 | 0.24 |
| Average slope (year) | -0.50 | 0.07 |
| Gaussian process standard deviation | 1.29 | 0.15 |
| *Gaussian process scale (year)* | ***1.61*** | ***0.36*** |
| Standard deviation of random intercept | 0.43 | 0.30 |
| Standard deviation of random slope | 0.23 | 0.06 |
| Correlation of random intercept and slope | -0.11 | 0.51 |
