# Supplementary material for: Visceral leishmaniasis: Spatiotemporal heterogeneity and drivers underlying the hotspots in Muzaffarpur, Bihar, India
Source: PLoS Negl Trop Dis. 2018 Dec 6;12(12):e0006888. doi: 10.1371/journal.pntd.0006888 (PMC6283467; doi:10.1371/journal.pntd.0006888)
Supplement: S2 Table — (DOCX) [file pntd.0006888.s002.docx]

| Year | Observed (I) | Expected | Z-score | P-value | Pattern |
| --- | --- | --- | --- | --- | --- |
| 2007 | 0.035 | -0.004 | 5.249 | <0.001 | *Clustered* |
| 2008 | 0.060 | -0.004 | 8.178 | <0.001 | *Clustered* |
| 2009 | 0.011 | -0.004 | 1.567 | 0.038 | *Clustered* |
| 2010 | 0.011 | -0.004 | 1.484 | 0.048 | *Clustered* |
| 2011 | 0.006 | -0.004 | 0.821 | 0.018 | *Clustered* |
| 2012 | 0.080 | -0.004 | 11.585 | <0.001 | *Clustered* |
| 2013 | 0.005 | -0.004 | 0.722 | 0.209 | Random |
| 2014 | 0.002 | -0.004 | 0.576 | 0.100 | Random |
| 2015 | 0.002 | -0.004 | 0.285 | 0.396 | Random |
