# Supplementary material for: Visceral leishmaniasis: Spatiotemporal heterogeneity and drivers underlying the hotspots in Muzaffarpur, Bihar, India
Source: PLoS Negl Trop Dis. 2018 Dec 6;12(12):e0006888. doi: 10.1371/journal.pntd.0006888 (PMC6283467; doi:10.1371/journal.pntd.0006888)
Supplement: S3 Table — (DOCX) [file pntd.0006888.s003.docx]

| Year | Cluster ID | Number of hamlets | Radius (km) | Population | Observed number of cases | Expected number of cases | Relative risk | P-value |
| --- | --- | --- | --- | --- | --- | --- | --- | --- |
| *Spatial hotspots* | | | | | | | | |
| 2007 | 1 | 6 | 0.5 | 1,857 | 25 | 2.3 | 15.0 | <0.001 |
|  | 2 | 39 | 1.9 | 7,961 | 24 | 7.1 | 4.9 | <0.001 |
|  | 3 | 2 | 0.1 | 343 | 4 | 0.2 | 20.9 | 0.025 |
| 2008 | 1 | 25 | 2.1 | 5,397 | 32 | 5.9 | 8.3 | <0.001 |
|  | 2 | 6 | 0.5 | 1,906 | 19 | 1.4 | 22.5 | <0.001 |
|  | 3 | 3 | 0.5 | 1,128 | 5 | 0.5 | 11.9 | 0.045 |
| 2009 | 1 | 2 | 0.3 | 786 | 6 | 0.3 | 28.1 | <0.001 |
|  | 2 | 39 | 1.5 | 10,066 | 10 | 2.7 | 5.9 | 0.047 |
| 2010 | 1 | 7 | 0.4 | 2,177 | 6 | 0.6 | 12.0 | 0.018 |
|  | 2 | 3 | 0.2 | 1,321 | 5 | 0.3 | 22.0 | 0.0047 |
| 2011 | 1 | 5 | 0.4 | 1,387 | 8 | 0.5 | 23.9 | <0.001 |
| 2012 | 1 | 8 | 0.8 | 1,828 | 28 | 0.9 | 86.0 | <0.001 |
| 2013 | 1 | 2 | 0.3 | 872 | 5 | 0.2 | 44.4 | <0.001 |
|  | 2 | 14 | 1.2 | 4,192 | 5 | 0.5 | 15.1 | 0.031 |
| 2014 | 1 | 6 | 0.6 | 1,510 | 4 | 0.2 | 41.9 | 0.0045 |
| 2015 | 1 | 11 | 0.9 | 2,627 | 3 | 0.1 | 54.5 | 0.038 |
| *Spatiotemporal hotspots* | | | | | | | | |
| 2007-2009 | 1 | 6 | 0.5 | 2,266 | 52 | 2.5 | 24.6 | <0.001 |
| 2011-2013 | 2 | 8 | 0.8 | 1,744 | 41 | 2.3 | 20.2 | <0.001 |
| 2007-2008 | 3 | 33 | 1.7 | 7,967 | 55 | 5.3 | 11.8 | <0.001 |
