# Supplementary figures and images for: Visceral leishmaniasis: Spatiotemporal heterogeneity and drivers underlying the hotspots in Muzaffarpur, Bihar, India
Source: PLoS Negl Trop Dis. 2018 Dec 6;12(12):e0006888. doi: 10.1371/journal.pntd.0006888 (PMC6283467; doi:10.1371/journal.pntd.0006888)

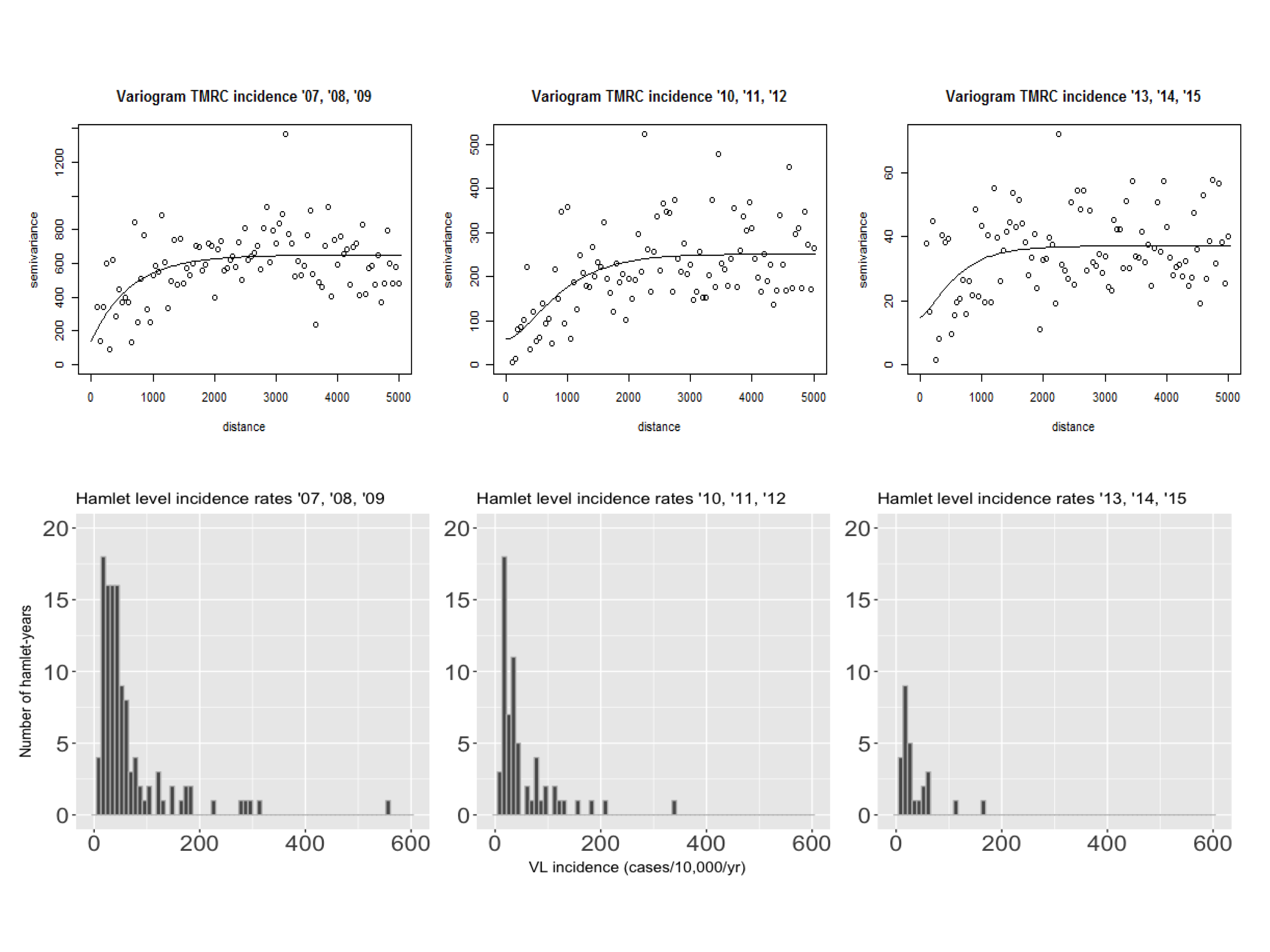

Supplement: S1 Fig — (TIF) [file pntd.0006888.s004.tif]

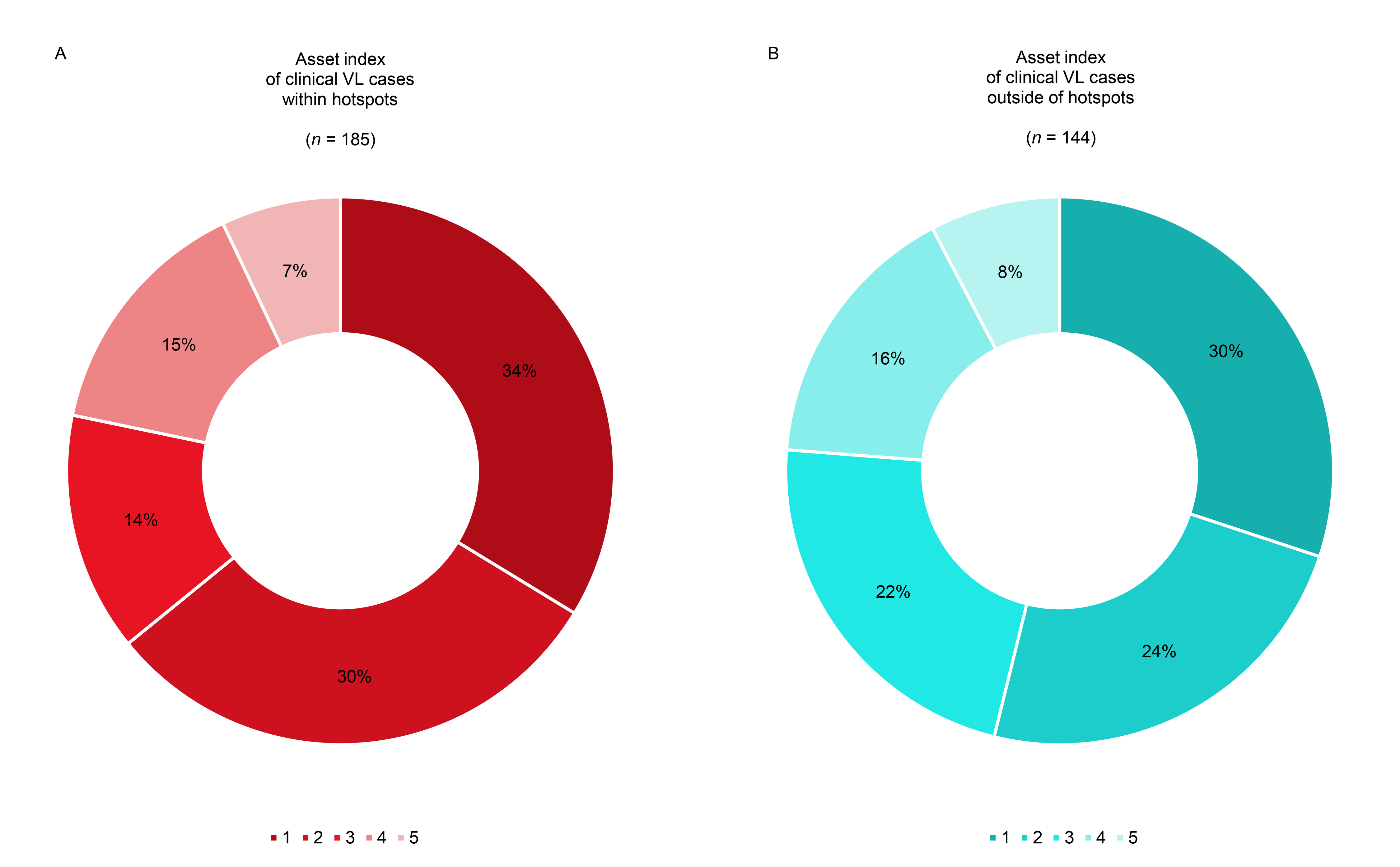

Supplement: S2 Fig — (TIF) [file pntd.0006888.s005.tif]

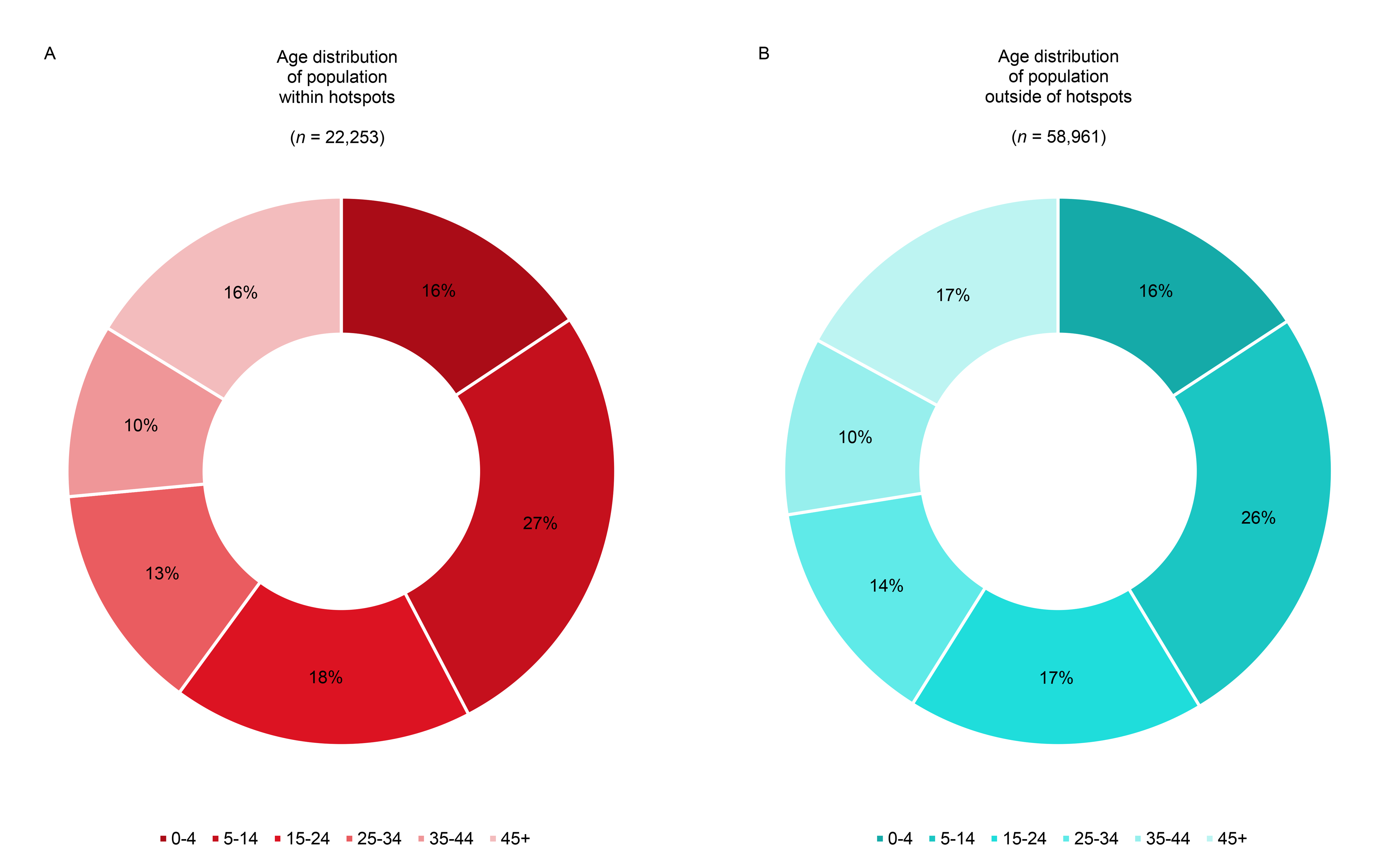

Supplement: S3 Fig — (TIF) [file pntd.0006888.s006.tif]

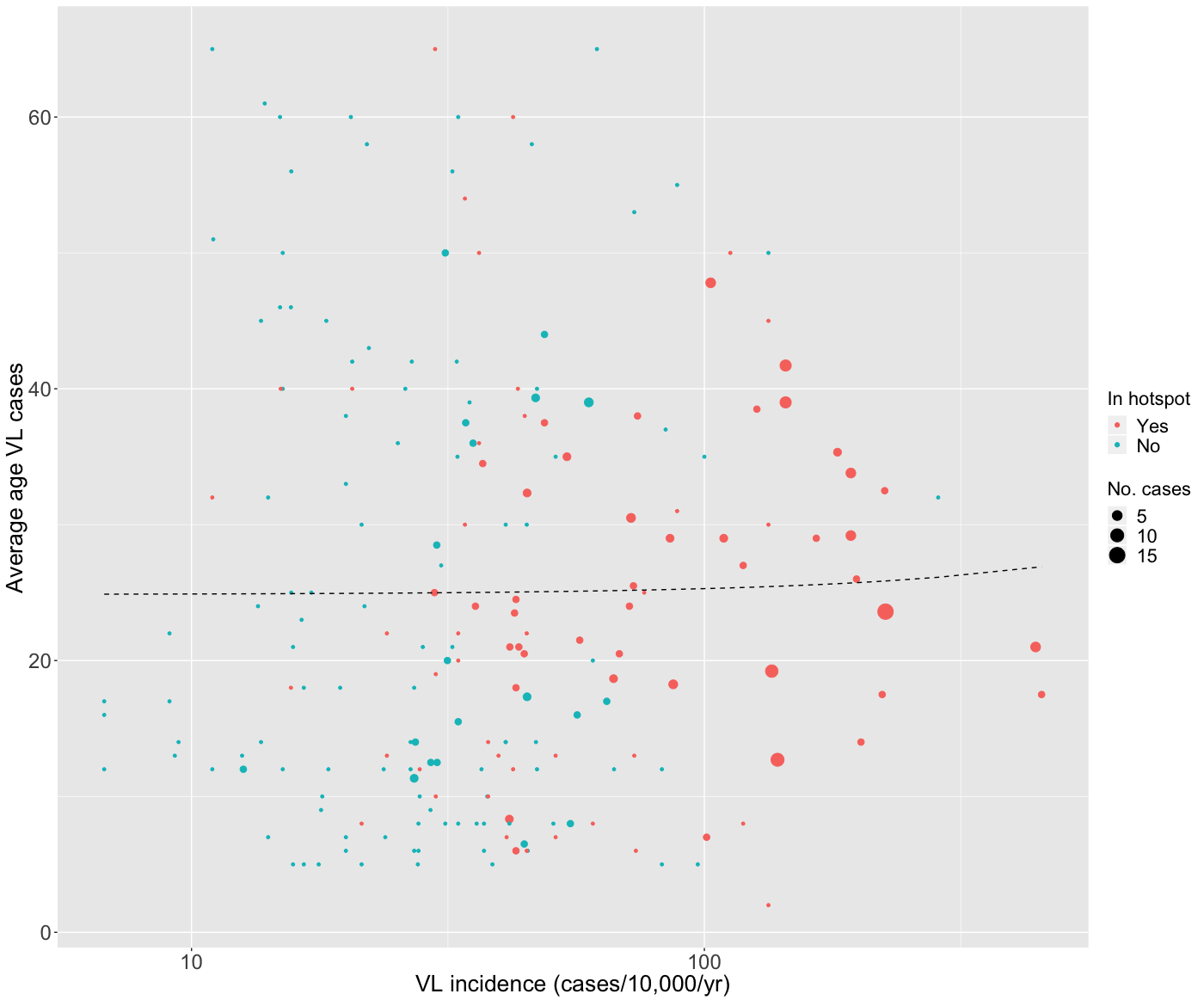

Supplement: S4 Fig — The black dashed line shows the regression line representing the association between the average age of VL cases per hamlet per year and the annual VL incidence at hamlet level. (TIF) [file pntd.0006888.s007.tif]

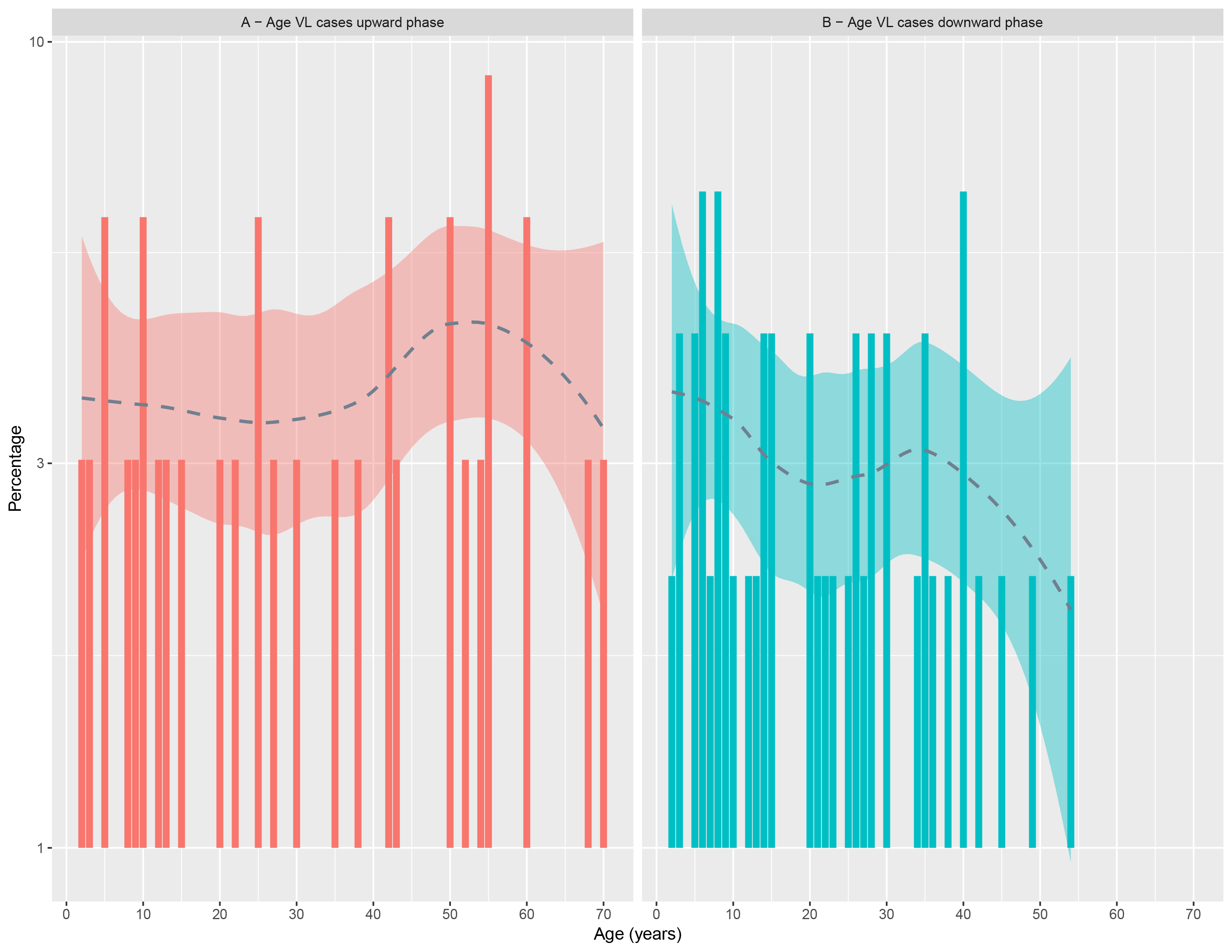

Supplement: S5 Fig — The bars represent the percentage (on a log scale) of reported VL cases. (TIF) [file pntd.0006888.s008.tif]
